# Digital Divide Among Homebound and Semi-Homebound Older Adults

**DOI:** 10.1177/07334648241292971

**Published:** 2024-10-16

**Authors:** Namkee G. Choi, Bryan Y. Choi, C. Nathan Marti

**Affiliations:** 1 12330 University of Texas at Austin, Austin, TX, USA; 2 6556 Philadelphia College of Osteopathic Medicine & Bay Health, Dover, DE, USA

**Keywords:** digital inequity, Blacks, Hispanics, social isolation, depression/anxiety, limited English proficiency

## Abstract

Using the 2022 National Health and Aging Trend Study data, we examined the digital divide among homebound and semi-homebound older adults. About 11% of older Medicare beneficiaries in 2022 were homebound or semi-homebound. Compared to non-homebound older adults, homebound older adults were significantly less likely to own a cellphone. Among those with any information and communication technology (ICT) device, homebound older adults were also less likely to have used email/texting and gone online. Older age, low income, dementia diagnosis, and moderate/severe depressive/anxiety symptoms contributed to the digital divide. Among homebound older adults, Hispanic older adults or those with limited English proficiency were less likely to have used ICT. To reduce the digital inequity among homebound older adults who are low-income, racial/ethnic minority, limited English proficient, and/or residents in non-metropolitan areas, social/structural inequities in accessibility and affordability, along with individual-level barriers, need to be addressed.

**What this paper adds**

• The digital divide is especially pronounced among Hispanic homebound older adults or those with limited English proficiency.
• Older age, low income, dementia diagnosis, moderate/severe depressive/anxiety symptoms, and non-metropolitan area residence are other contributors to the digital divide.
• The digital divide among homebound older adults suggests the link between social engagement and digital engagement.

**Applications of study findings**

• It is imperative that homebound older adults become digitally connected and skilled at navigating the Internet for health-related communications and care delivery.
• To reduce the digital divide among homebound older adults, social/structural inequities in accessibility and affordability need to be addressed.
• Helping homebound older adults who are not information and communication technology (ICT) users adopt ICT will likely facilitate equitable access to healthcare and social services and improve their social integration.

## Introduction

Homebound older adults are often defined as those who rarely/never left home in the past month, and semi-homebound older adults are defined as those who only left the home with assistance or had difficulty or needed help leaving the home. With progression of aging, more older adults become homebound over time. In their study of Medicare beneficiaries age 65+, Xiang et al. (2020) found that 8.3% were chronically homebound (i.e., rarely/never went out) and 26.2% became homebound over the 7-year (2011–2017) study period. Another study of Medicare beneficiaries showed that 16.2% were homebound or semi-homebound in 2019 (Ankuda et al., 2022). The proportions of homebound or semi-homebound older adults appear to fluctuate over time in the U.S. (Ankuda et al., 2021), but they are a significant share of the older-adult population in a rapidly aging society.

Research has shown that older age, female sex, racial/ethnic minority status, not having a college degree, low income, chronic medical conditions, functional and cognitive impairments, and/or depression/anxiety were associated with increased risk of chronic or a new onset homebound state (Ankuda et al., 2021, 2022; Ornstein et al., 2015, 2020; Xiang et al., 2020; Yang et al., 2021). Compared to non-homebound peers, homebound older adults also reported significantly higher levels of social isolation and loneliness (Cudjoe et al., 2022; Ezeokonkwo et al., 2021). A study of Medicare beneficiaries over seven years also found that social isolation increased risk of homebound progression among those without dementia at baseline (Yang et al., 2021).

Information and communication technologies (ICT) have been heralded as an important tool that can promote health among older adults by facilitating their access to health information and healthcare services, social interactions, and daily living (Gunnes et al., 2024; Nordin et al., 2021; Thangavel et al., 2022; Wilson et al., 2022, 2024). Internet use has been found to be associated with decreased social isolation and loneliness and increased social contacts among older adults (Balki et al., 2023; Casanova et al., 2021; Yu et al., 2021; Zhang et al., 2022). The COVID-19 pandemic helped many older adults adopt ICT when in-person interactions with family/friends, social engagement, and healthcare services had to be curtailed and replaced with technology-based remote contact (Choi et al., 2022).

While the generational gap in ICT ownership and use has been decreasing, the digital divide among older adults by socioeconomic and physical, mental, and cognitive health statuses continues since older age (e.g., 75+ years), racial/ethnic minority status, and lower education and household income remain persistent correlates of the digital divide (Arcury et al., 2020; Chan et al., 2023; Kebede et al., 2022; Schroeder et al., 2023; Wilson et al., 2022). Homebound older adults, who tend to include higher proportions of Blacks and Hispanics and have lower income and greater degrees of functional and cognitive impairments than their non-homebound peers, are less likely to own an ICT device and go online (Choi & Dinitto, 2013). However, digital divide among homebound older adults post-COVID-19 pandemic has not been examined. The COVID-19 pandemic may have widened the digital divide as low-income older adults with poor health may have been further left out in the rapidly digitalized society (Cheung et al., 2023; Litchfield et al., 2021). It is important to examine continuing digital divide and associated factors among homebound older adults with respect to ICT device ownership and use in post-COVID-19 pandemic times.

### The Digital Divide: Barriers to ICT Use Among Homebound Older Adults

The digital divide is multifaceted and refers to the gaps in accessibility, affordability, digital literacy and skills, quality, and relevance of digital technologies among different population groups (Choxi et al., 2022; Litchfield et al., 2021; Mubarak & Suomi, 2022). Low-income older adults in general are subjected to the digital divide due to the high cost of ICT device and Internet subscription (Choi & Dinitto, 2013). Broadband services may also be unavailable or costlier in rural locations (Bell et al., 2023).

Given their limited mobility, access to ICT devices and the Internet at a place of residence is critical to ICT use among homebound older adults. However, those who lack digital literacy and skills may also experience fewer opportunities to learn the skills if they do not have someone to assist them at home (Berkowsky et al., 2018; Choi et al., 2022). A study found that for their first video telehealth primary care visit during the early months of the COVID-19 pandemic, over 82% of homebound older adults required assistance from a family member and/or paid caregiver to complete the visit (Kalicki et al., 2021). The same study also found that among those who had not used telehealth, 27% were deemed “unable to interact over video” for reasons including cognitive or sensory impairment and 14% lacked access to a caregiver who could assist them with technology (Kalicki et al., 2021). Whilst ICT use can reduce social isolation and loneliness as described, social isolation can be a significant barrier to digital literacy and skills acquisition. Lack of digital literacy may also be affected by limited English proficiency (LEP) (Casillas et al., 2018).

Capabilities are also often related to physical and mental health conditions (Kebede et al., 2022). In Choi and DiNitto’s study (2013), low-income homebound older adults reported body pain, unsteady hands, vision and other sensory problems, difficulty sitting for a long time, difficulty concentrating due to chronic fatigue and other health conditions as barriers to using the Internet and computers. In terms of quality, discounted telephone services (i.e., lifeline program; https://www.fcc.gov/general/lifeline-program-low-income-consumers) often have limited functionalities. Studies have also shown that perceived relevance (value, usefulness, advantages of a new technology), comfort with using new technology, and perceived impact on quality of life are important predictors of older adults’ willingness to adopt technology (Berkowsky et al., 2018; Loizos et al., 2023; Moxley et al., 2022; Sharit et al., 2021). Homebound older adults with multiple barriers to adopting ICT may not see the value of ICT use if the barriers deemed too difficult to surmount. In an initiative of an urban primary care visiting doctor program for homebound older adults during the pandemic, less than half of those who were provided cellular-enabled video telehealth devices used them and one third of the nonusers returned them (Loizos et al., 2023). The authors noted that telephone visits were the predominant mode of communication and video visits were least used mode.

### Research Questions

In this study, using a nationally representative older-adult sample in 2022, we first examined the associations of ICT device (cellphone and computer/tablet) ownership and ICT (email/texting and online) use with homebound or semi-homebound state along with demographic characteristics (age, gender, race/ethnicity, and marital status); residential type (own home vs. care community) and location (metropolitan vs. nonmetropolitan); affordability indicators (education and income); social isolation indicators (no one to talk to; driving status); and health-related capability indicators (dementia diagnosis, number of other diagnosed medical conditions, activity-limiting pain; depression/anxiety). The study hypotheses were that compared to their non-homebound peers, homebound, and semi-homebound older adults were less likely to own a cell phone (H1a) and a computer/tablet (H1b) and to use email/texting (H2a) and go online (H2b), even after controlling for these other covariates. Second, we examined the correlates of ICT use among homebound older adults. The study hypotheses were that older age, racial/ethnic minority status or LEP, and capability indicators will be associated with a lower likelihood of email/texting (H3a) and online use (H3b) among homebound older adults.

## Materials and Methods

### Data Source

Data came from the 2022 U.S. National Health and Aging Trends Study (NHATS). NHATS collects data annually from a nationally representative panel of Medicare beneficiaries (age 65+) on their physical, functional, cognitive, and sensory capacity, social, physical, and technological environments, and participation in valued activities. The initial NHATS sample persons were interviewed in 2011, and the first and second replenishment samples were added in 2015 and 2022, respectively (Freedman et al., 2023). The 2022 NHATS data were collected in in-person interviews. In this study, we focused on 5593 sample persons, representing approximately 49.4 million Medicare beneficiaries age 65+, who were living in their own homes or residential care communities (but not in nursing homes) and self-reported data (i.e., no proxy interview). We excluded proxy-interviewed sample persons (*n* = 241) to ensure that all data were self-reported. This study, based on de-identified public-use data, was exempt from the authors’ institutional review board review.

### Measures

#### Homebound and Semi-Homebound State

Respondents were asked, “In the last month, how often did you leave your building/home to go outside?” Response categories were everyday (7 days a week), most days (5–6 days a week), some days (2–4 days a week), rarely (once a week or less), and never. We categorized rarely or never leaving home as homebound state. Semi-homebound state was assigned when the respondents, even though they left home at least some days, reported that they had a lot of difficulty going outside by themselves, rarely/never went outside by themselves, or had to stay in because there was no one to help them go outside.

#### ICT Device Ownership and Use

ICT devices were a working cellphone, a working desktop or laptop computer, and a tablet (excluding eBook readers) at home/apartment/unit/suite/other (yes or no). ICT use in the past month was assessed with (1) ever sent messages by email or texting and (2) ever used the Internet or online for any other reason than email/texting (yes or no). Online activities (e.g., shopping, healthcare communication, and visiting social network sites) were presented for descriptive purposes only.

#### Sociodemographic Factors

These included age in 2022 (65–74, 75–84, 85+), gender (female vs. male), race/ethnicity (non-Hispanic White, non-Hispanic Black, Hispanic, all other), marital status (married/partnered, divorced/separated, widowed, never married), residential type (own home vs. care community) and location (metropolitan area vs. other), and LEP (interviewed in Spanish or other languages than English).

#### Affordability Indicators

These were education (bachelor’s degree vs. no degree) and income (<$43,000, $43,000–<$84,000, ≥$84,000, missing).

#### Social Isolation Indicators

These were self-reports of having no one to talk to (yes vs. no) and driving a car (yes vs. no).

#### Health-Related Capability Indicators

These included dementia diagnosis, number of diagnosed medical conditions excluding dementia (0–9; arthritis, cancer, diabetes, heart attack or heart disease, hypertension, lung disease, osteoporosis, stroke, and other serious illness), activity limiting pain in the past month (yes vs. no), and depression/anxiety symptoms in the past month. In NHATS, depression/anxiety symptoms were assessed with the Patient Health Questionnaire-4 (PHQ-4) (Kroenke et al., 2009). The PHQ-4 includes the first two items (PHQ-2; had little interest or pleasure in doing things, and felt down, depressed, or hopeless) from the 9-item PHQ-9 for depression (Kroenke et al., 2003) and the first two items (GAD-2; felt nervous, anxious, or on edge, and have been unable to stop or control worrying) from the 7-item Generalized Anxiety Disorder Scale (Spitzer et al., 2006). Responses to each PHQ-4 item were based on a 4-point scale (0 = not at all; 1 = several days; 2 = more than half the days; 3 = nearly every day), with the total score ranging from 0 to 12 (0–2 indicating normal, 3–5 mild, and 6–12 moderate/severe symptoms; Kroenke et al., 2009). Unweighted Cronbach’s alpha for the PHQ-4 for the study sample was .74.

### Analysis

All analyses were conducted with Stata/MP 18’s svy function (College Station, TX) to account for NHATS’s stratified, multistage sampling design. First, we used *χ*^2^ tests, ANOVA, and *t* test to compare non-homebound, homebound, and semi-homebound older adults in terms of demographic and other characteristics and ICT device ownership. Second, we used *χ*^2^ tests to compare ICT use among the three groups of older adults who owned any ICT device. Third, for H1a and H1b (association between ICT device ownership and homebound state) testing, we fitted two logistic regression models to examine the associations between homebound/semi-homebound states and cellphone and computer/tablet ownerships, respectively. For H2a and H2b (association between ICT use and homebound state) testing, we focused on those who owned an ICT device and fitted two generalized linear models (GLMs) for a Poisson distribution with a log link, with email/texting use and online use, respectively, as the dependent variable in each model. Sociodemographic characteristics, affordability, social isolation, and capability factors were entered as covariates. We fit GLMs rather than a logistic regression model because odds ratios exaggerate the true relative risk to some degree when the event (i.e., ICT use among ICT device owners) is a common (i.e., >10%) occurrence (Grimes & Schulz, 2008). Fourth, for H3a and H3b (correlates of ICT use among homebound older adults), we also fitted two GLMs for a Poisson distribution with a log link, with email/texting use and online use, respectively, as the dependent variable in each model. Because of the high collinearity between race/ethnicity and LEP (i.e., variance inflation factor >2.50; Allison, 2012), we used two separate models, one including race/ethnicity and the other including LEP. Logistic regression results are presented as adjusted odds ratios (aORs) with 95% confidence intervals (CI), and GLM results are reported as incidence rate ratios (IRRs) with 95% CI. Significance was set at *p <* .05.

## Results

### Characteristics of Older Adults by Homebound Status

Table 1 shows that 4.9% (95% CI = 4.2–5.6) of the Medicare beneficiaries were homebound and 6.5% (95% CI = 5.5–7.7) were semi-homebound in the preceding month. Compared to non-homebound peers, homebound and semi-homebound older adults had higher proportions of those age 85+, females, racial/ethnic minorities, those with LEP, care community residents, and those with income <$43,000, but lower proportions of married individuals, college graduates, and drivers. Additional analysis showed that 50.4% of all Hispanic older adults were interviewed in Spanish. All health status/capability indicators show that homebound and semi-homebound older adults were significantly worse off than their non-homebound peers. Comparison between homebound and semi-homebound groups show that the homebound group had higher proportions of those age 85+ and with LEP but lower proportions of drivers and those with activity-limiting pain.

**Table 1.** Characteristics by Homebound State.

	Not Homebound or Semi-Homebound	Homebound	Semi-Homebound	*p* Among All 3 Groups	*p* Between Homebound and Semi-Homebound
*N*	4742	416	435		
% of all (95% CI)	88.6 (87.2–89.9)	4.9 (4.2–5.6)	6.5 (5.5–7.7)		
Age group (years, %)				<.001	.038
65–74	58.2	40.3	53.0		
75–84	33.2	38.0	29.1		
85+	8.6	21.7	17.9		
Female (%)	53.0	73.6	65.0	<.001	.072
Race group				<.001	.051
Non-Hispanic White	79.8	60.8	70.9		
Non-Hispanic Black	8.2	14.3	10.7		
Hispanic	7.2	19.6	12.9		
Other	4.8	5.3	5.5		
Married/partnered (%)	61.7	33.1	42.6	<.001	.074
Care community residence (%)	3.2	11.6	8.3	<.001	.267
Metropolitan area residence (%)	83.1	85.3	84.4	.686	.802
College (BA/BS) degree (%)	38.8	18.8	27.5	<.001	.065
Total income (%)				<.001	.467
<$43,000	35.6	65.4	63.7		
$43,000–<$84,000	25.9	11.6	14.7		
≥$84,000	27.6	8.4	11.1		
Missing	10.9	14.6	10.5		
Limited English proficiency (%)	3.9	19.1	9.9	<.001	.006
No one to talk to (%)	4.5	8.9	5.4	.131	.334
Drives a car (%)	88.3	28.7	45.1	<.001	.004
Dementia diagnosis (%)	1.9	8.1	7.2	<.001	.719
No. of medical conditions (M, SE)	2.41 (0.03)	3.53 (0.12)	3.58 (0.10)	<.001	.764
Activity-limiting pain (%)	27.9	60.9	71.1	<.001	.026
Depression/anxiety symptoms (%)				<.001	.279
Normal	77.3	46.2	38.9		
Mild	16.9	30.3	34.5		
Moderate/severe	5.9	23.5	26.6		
ICT device ownership (%)					
Cell phone	96.4	84.7	90.9	<.001	.014
Computer	80.1	58.7	66.9	<.001	.089
Tablet	58.8	42.7	49.0	<.001	.141
Computer or tablet	86.5	70.0	74.5	<.001	.241
Any of the above					

*Note.* Probability values were calculated based on Pearson’s *χ*^2^ tests, ANOVA, and *t* tests.

Table 1 also shows that compared to 4.6% of non-homebound older adults, 15.3% of homebound and 9.1% of semi-homebound older adults did not own a working cellphone. Compared to 13.5% of non-homebound older adults, 30.0% of homebound and 25.5% of semi-homebound older adults did not own a computer or a tablet.

### Types of ICT Use Among Those With ICT Device

Table 2 shows that of the three groups, homebound older adults were least likely to have used email/texting (51.7%) and gone online (45.6%), followed by semi-homebound older adults (66.6% and 60.2%, respectively, compared to 84.1% and 77.6%, respectively, among non-homebound peers). For all three groups of online users, grocery/other personal item shopping, bill paying and banking, and visiting social network sites were the top three online activities in the past month. Significantly lower proportions of homebound (20.5%) and semi-homebound (29.4%) than non-homebound (43.3%) older adults had telehealth visits.

**Table 2.** ICT Use Among ICT Device Owners by Homebound State (%).

	Not Homebound or Semi-Homebound	Homebound	Semi-Homebound	*p* Among All 3 Groups	*p* Between Homebound and Semi-Homebound
*N*	4623	376	399		
%	89.1	4.6	6.3		
Email/texting	84.1	51.7	66.6	<.001	.001
Online use	77.6	45.6	60.2	<.001	.010
Grocery or other personal item shopping	47.9	31.2	38.0	<.001	.187
Paying bills or banking	54.6	30.2	36.6	<.001	.275
Ordering or refilling prescription medications	31.4	20.7	28.6	.045	.163
Telehealth visit	43.3	20.5	29.4	<.001	.062
Contacting medical providers	26.8	19.6	28.7	.187	.033
Obtaining information on health conditions	23.4	7.0	17.8	<.001	.007
Handling Medicare or other health insurance matters	35.8	22.0	23.6	<.001	.723
Visiting social network sites	47.0	26.4	38.1	<.001	.021
Video calls with family/friends	37.7	21.1	28.5	<.001	.128

*Note.* Probability values were calculated based on Pearson’s *χ*^2^ tests.

### ICT Device Ownership and Homebound State: Multivariable Logistic Regression Analyses

The second and third columns of Table 3 show that homebound older adults compared to non-homebound peers had lower odds of cellphone ownership (aOR = 0.58, 95% CI = 0.34–0.98). However, homebound state was not associated with computer/tablet ownership. The results provide support for H1a for homebound older adults only.

**Table 3.** Correlates of ICT Device Ownership (Logistic Regression) and ICT Use Among ICT Device Owners (Generalized Linear Modeling).

	ICT Device Ownership		ICT Use	
	Cellphone Ownership aOR (95% CI)	Computer/Tablet Ownership aOR (95% CI)	Email/Texting Use Versus Nonuse IRR (95% CI)	Online Use Versus Nonuse IRR (95% CI)
Homebound state: versus not homebound or semi-homebound				
Homebound	0.58 (0.34–0.98)*	1.12 (0.79–1.58)	0.81 (0.73–0.89)***	0.81 (0.70–0.94)**
Semi-homebound	0.80 (0.53–1.23)	0.93 (0.60–1.44)	0.94 (0.87–1.02)	0.96 (0.83–1.09)
Age group: versus 65–74 years				
75–84	0.51 (0.33–0.80)**	0.67 (0.51–0.88)**	0.88 (0.85–0.91)***	0.89 (0.85–0.93)***
85+	0.20 (0.13–0.32)***	0.44 (0.34–0.57)***	0.66 (0.61–0.72)***	0.68 (0.62–0.76)***
Female (%)	1.56 (1.01–2.42)*	1.37 (1.09–1.73)**	1.09 (1.06–1.13)***	1.04 (0.99–1.08)
Race group: Versus non-Hispanic white				
Non-Hispanic Black	1.46 (0.80–2.66)	0.56 (0.45–0.69)***	0.83 (0.78–0.88)***	0.71 (0.66–0.76)***
Hispanic	1.73 (0.93–3.22)	0.41 (0.32–0.54)***	0.74 (0.66–0.82)***	0.68 (0.60–0.77)***
Other	1.43 (0.76–2.70)	0.67 (0.35–1.28)	0.93 (0.85–1.03)	0.80 (0.69–0.91)**
Married/partnered (%)	1.03 (0.63–1.69)	1.66 (1.26–1.19)**	0.98 (0.94–1.01)	1.03 (0.98–1.08)
Own residence versus care community residence	1.22 (0.76–1.96)	1.53 (1.00–2.34)	0.92 (0.82–1.03)	0.92 (0.82–1.03)
Metropolitan versus nonmetropolitan area residence	1.31 (0.77–2.22)	1.55 (1.09–2.21)*	1.07 (1.02–1.12)**	1.15 (1.06–1.25)**
College (BA/BS) versus no college degree	1.66 (1.02–2.71)*	2.79 (1.86–4.19)***	1.15 (1.12–1.18)***	1.20 (1.15–1.26)***
Income: versus <$43,000				
$43,000–<$84,000	1.83 (1.27–2.65)**	3.59 (2.59–4.96)***	1.11 (1.06–1.15)***	1.20 (1.13–1.27)***
≥$84,000	2.24 (1.25–4.04)**	5.38 (3.26–8.88)***	1.13 (1.08–1.18)***	1.22 (1.16–1.28)***
Missing	1.03 (0.66–1.60)	1.45 (1.03–2.03)*	0.98 (0.92–1.05)	1.03 (0.94–1.13)
No one versus someone to talk to	1.35 (0.73–2.50)	0.78 (0.49–1.24)	0.94 (0.85–1.04)	0.89 (0.76–1.03)
Drives versus does not drive a car	3.62 (2.27–5.77)***	1.73 (1.33–2.25)***	1.28 (1.21–1.37)***	1.24 (1.15–1.33)***
Dementia diagnosis versus no diagnosis	0.49 (0.26–0.92)*	0.68 (0.40–1.16)	0.77 (0.67–0.90)**	0.78 (0.63–0.98)*
No. of medical conditions	1.11 (0.99–1.23)	1.04 (0.95–1.14)	1.00 (0.99–1.01)	1.01 (0.99–1.02)
Activity-limiting pain versus no activity-limiting pain	1.01 (0.71–1.43)	1.13 (0.90–1.40)	1.04 (0.99–1.08)	1.07 (1.02–1.12)**
Moderate/severe versus normal/mild depression/anxiety symptoms	1.38 (0.79–2.39)	0.66 (0.46–0.97)*	0.95 (0.89–1.01)	0.89 (0.81–0.98)*
Model statistics	*N* = 5593; design df = 56; F (21,36) = 26.47; *p* < .001	*N* = 5593; design df = 56; F (21,36) = 17.00; *p* < .001	*N* = 5398; design df = 56	*N* = 5398; design df = 56

*p < .05. **p < .01. ***p < .001.

Of the covariates, the 75–84 and the 85+ age groups (vs. 65–74 age group), had lower odds of cellphone and computer/tablet ownership, but females, married people, those with a college education, those with income ≥$43,000 (vs. income <$43,000), and those who drove a car had higher odds. Race/ethnicity was not associated with cellphone ownership; however, Blacks and Hispanics (vs. non-Hispanic Whites) had significantly lower odds of computer/tablet ownership. Metropolitan, relative to non-metropolitan, area residence was also associated with higher odds of computer/tablet ownership. Of the capability indicators, only dementia diagnosis (lower odds of cellphone ownership) and moderate/severe depression/anxiety (lower odds of computer/tablet ownership) were significant. Residential type, number of chronic health conditions, activity-limiting pain, and having no one to talk to were not significantly associated with cellphone or computer/tablet ownership.

### ICT Use and Homebound State: GLM Results

The fourth and fifth columns of Table 3 show that among ICT device owners, homebound older adults, compared to their non-homebound peers, had lower likelihood of email/texting (IRR = 0.81, 95% CI = 0.73–0.89), and any online use (IRR = 0.81, 95% CI = 0.70–0.94). However, semi-homebound older adults did not differ from their non-homebound peers. The results provide support for H2a and H2b for homebound older adults only.

Of the covariates, older ages (75–84 and 85+), racial/ethnic minorities, and dementia diagnosis were associated with a lower likelihood of email/texting and online use, while metropolitan area residence, driving a car, college education, and income ≥$43,000 were associated with a higher likelihood. Female sex was associated with a higher likelihood of email/texting use, and activity-limiting pain was associated with a higher likelihood of online use, but moderate/severe depression/anxiety was associated with a lower likelihood of online use.

### ICT Use Among Homebound Older Adults

The second and third columns of Table 4 show that older ages, Hispanic ethnicity (IRR = 0.51, 95% CI = 0.33–0.79) or LEP (IRR = 0.43, 95% CI = 0.28–0.65), and dementia diagnosis were associated with a lower likelihood, while more chronic medical conditions were associated with a higher likelihood of email/texting use. The fourth and fifth columns show that Hispanic ethnicity (IRR = 0.56, 95% CI = 0.36–0.86) or LEP (IRR = 0.59, 95% CI = 0.40–0.89) were associated with a lower likelihood, while metropolitan location and driving a car were associated with a higher likelihood of online use. The results support H3a and H3b with regard to age, minority status, LEP, and/or capability indicators (dementia diagnosis in particular) as correlates of homebound older adults’ ICT use.

**Table 4.** Correlates of Email/Testing and Online Use Among Homebound Older Adults With ICT Device: Generalized Linear Modeling Results.

	Email/Texting Use Versus Nonuse IRR (95% CI)		Online Use Versus Nonuse IRR (95% CI)	
Age group: versus 65–74 years				
75–84	0.74 (0.58–0.95)*	0.78 (0.61–0.99)*	0.87 (0.64–1.18)	0.91 (0.66–1.24)
85+	0.61 (0.41–0.90)*	0.66 (0.45–0.96)*	0.79 (0.48–1.30)	0.86 (0.53–1.40)
Female (%)	1.10 (0.81–1.49)	1.08 (0.80–1.44)	1.09 (0.75–1.58)	1.09 (0.75–1.59)
Race group: versus non-Hispanic white				
Non-Hispanic Black	0.84 (0.63–1.12)		0.69 (0.46–1.03)	
Hispanic	0.51 (0.33–0.79)**		0.56 (0.36–0.86)**	
Other	1.05 (0.48–2.31)		1.06 (0.52–2.15)	
Spanish/other language versus English interview		0.43 (0.28–0.65)***		0.59 (0.40–0.89)*
Married/partnered (%)	0.92 (0.68–1.25)	0.94 (0.68–1.28)	1.10 (0.75–1.61)	1.13 (0.78–1.66)
Own residence versus care community residence	1.20 (0.61–2.35)	1.11 (0.60–2.06)	1.38 (0.60–3.15)	1.25 (0.56–2.78)
Metropolitan versus nonmetropolitan area residence	1.00 (0.68–1.46)	1.01 (0.70–1.45)	1.45 (1.01–2.10)*	1.39 (0.96–2.01)
College (BA/BS) versus no college degree	1.22 (0.91–1.65)	1.22 (0.93–1.59)	1.28 (0.89–1.84)	1.33 (0.93–1.89)
Income				
$43,000–<$84,000	1.00 (0.73–1.38)	0.98 (0.72–1.32)	1.34 (0.88–2.05)	1.33 (0.87–2.02)
≥$84,000	1.44 (0.96–2.17)	1.32 (0.87–2.00)	1.23 (0.71–2.14)	1.19 (0.68–2.10)
Missing	0.89 (0.56–1.42)	0.85 (0.54–1.34)	1.07 (0.63–1.81)	1.00 (0.61–1.64)
No one versus someone to talk to	0.82 (0.48–1.41)	0.82 (0.48–1.39)	1.11 (0.62–2.00)	1.10 (0.60–2.04)
Drives versus does not drive a car	1.17 (0.92–1.48)	1.28 (0.98–1.67)	1.44 (0.98–2.10)	1.56 (1.07–2.28)*
Dementia diagnosis versus no diagnosis	0.23 (0.06–0.91)*	0.25 (0.06–0.93)*	0.45 (0.15–1.38)	0.48 (0.16–1.42)
No. of medical conditions	1.11 (1.02–1.21)*	1.10 (1.01–1.20)*	1.07 (0.97–1.17)	1.06 (0.97–1.17)
Activity-limiting pain versus no activity-limiting pain	1.19 (0.77–1.82)	1.22 (0.81–1.83)	1.28 (0.86–1.92)	1.32 (0.88–1.98)
Moderate/severe versus normal/mild depression/anxiety symptoms	0.71 (0.53–0.96)*	0.74 (0.55–0.99)*	0.72 (0.49–1.06)	0.75 (0.51–1.12)
Model statistics	*N* = 376; design df = 51	*N* = 376; design df = 51	*N* = 376; design df = 51	*N* = 376; design df = 51

*p < .05. **p < .01. ***p < .001.

## Discussion

This study found that 11% of older Medicare beneficiaries in 2022 were homebound or semi-homebound. As expected, these older adults, compared to their non-homebound peers, included higher proportions of those who were age 85 years and older, female, racial/ethnic minority, not married, low-income, and socially isolated. They also had more chronic medical conditions and included higher proportions of those with dementia diagnosis, activity-limiting pain, and depressive/anxiety symptoms.

Descriptive statistics show that compared to non-homebound older adults, lower proportions of homebound and semi-homebound older adults owned a cellphone or a computer/tablet and have used email/texting and gone online. In multivariable analyses controlling for sociodemographics and indicators of affordability, social isolation, and health-related capabilities, homebound state was associated with lower odds of cellphone, but not computer/tablet, ownership. Among those who own an ICT device, homebound older adults had a lower likelihood of email/texting and online use. On the other hand, those who were semi-homebound did not significantly differ from non-homebound older adults on ICT ownership and use in multivariable analyses.

No difference between semi-homebound and non-homebound older adults in ICT device ownership and use suggests that going outside their home with others’ help despite their mobility difficulty increases digital engagement. The link between social or other engagement outside the home and digital engagement can also be deduced from the high rates of digital activities among non-homebound older adults as well as by the significant positive association between driving status and ICT ownership and use. Those who helped semi-homebound older adults to leave home may also have been able to help these older adults with ICT use. On the other hand, social isolation may be a contributing factor to the digital divide among homebound older adults.

The older ages and a higher proportion of Black and Hispanic older adults among homebound older adults have also likely contributed to their lower likelihood of ICT use. A previous study based on the 2018 NHATS found that Black and Hispanic older adults were less likely to own a cellphone and a computer/tablet (Suntai & Beltran, 2023). Our study shows that the racial/ethnic disparity in cellphone ownership continued in 2022, even after the COVID-19 pandemic. Affordability was likely a significant factor, as cellphone and Internet access could have been financially out of reach for low-income homebound older adults (Choi & Dinitto, 2013). As mentioned, in rural areas, affordability may have been an even more important factor. Previous studies also found that Black and Hispanic than non-Hispanic white older adults tended to prefer using non-digital modalities and had a higher level of distrust in digital modalities (Gordon & Hornbrook, 2016; Johnson, 2023; Mitchell et al., 2019).

Our study also shows that the digital divide among a significant proportion of Hispanic older adults likely stems from their LEP. A study based on the 2011 and 2021 NHATS found the digital divide between Hispanic and non-Hispanic White older adults have been narrowing (Wang et al., 2024); however, LEP was seldom examined as a contributor to the digital divide among racial/ethnic minority older adults. A previous study of a group health cooperative found that patients with LEP had lower use of the Internet medication refill system and lower medication adherence than patients with English proficiency (Casillas et al., 2018). Primary care physicians involved in post-pandemic virtual care also observed that patients with LEP were particularly affected by inequity in virtual care access (Guetterman et al., 2023).

Of the capability factors, along with dementia, moderate/severe depression/anxiety was a significant barrier to ICT use among all older adults well as among homebound older adults. This is consistent with a recent study’s finding that older email/texting and SNS users had a lower likelihood of depressive/anxiety than never users and discontinued users (Choi et al., 2024). Depression/anxiety may have sapped motivation for and interest in learning or continuing to use technology to connect and interact with others. On the other hand, we found that the number of medical conditions per se was not a significant determinant, and activity-limiting pain was a contributing factor to online use. This underscores the importance of paying attention to mental health conditions when assisting older adults with their digital engagement.

In sum, this study shows that the digital divide among homebound older adults is rooted in both individual vulnerability factors, such as cognitive impairment and depression/anxiety, and social/structural inequities that many low-income older adults have been persistently subjected to. To assist homebound older adults who are low-income and racial/ethnic minority and/or residents in non-metropolitan areas to use ICT, social/structural inequities in accessibility and affordability need to be addressed.

There are some study limitations due to data constraints: (1) only correlation, not causation, can be derived from cross-sectional survey data, (2) all data were self-reported and may have been subjected to recall and social desirability bias, and (3) the data did not include reasons for not owing ICT devices and choosing not to use ICT.

Despite these limitations, the study findings indicate the need to invest resources to close the digital divide among homebound older adults. As healthcare and social service systems have become digitalized, it is imperative that all older adults become digitally connected and skilled at navigating the Internet for health-related communications and care delivery, searching for aging services resources, and applying for benefits. Tele-delivered health and mental health and aging services are likely to become the norm, not an exception, in the near future (Hagi et al., 2023). Equitable access to healthcare and social services and improved social integration are essential for overall well-being in late life. Specifically, this study shows that along with simplifying access to telehealth and online social services for older adults, diverse programming (e.g., Spanish and other languages) and easily accessible ICT training services are needed for digitally excluded older adults. Social service providers need to connect these older adults to training programs like Older Adults Technology Services (OATS, 2024) and foster community programs where tech-savvy peers or younger generations can provide ongoing digital support. Social service providers also need to help low-income older adults find resources for accessing affordable technology programs, working with organizations that provide grants or equipment donations to older adults. The Federal Communications Commission’s Affordable Connectivity Program that provided free or discounted high-speed Internet for millions of eligible households (including 4 million older adults) ended in June, 2024 due to a lack of additional funding from Congress (Congressional Research Service, 2024). Congress needs to pass legislation to extend the program.

For homebound older adults suffering from depression/anxiety, research is needed to find ways to influence their motivation and intention to use digital technologies. More research is needed to explore older adults’ perceptions and motivations about using different types of digital technologies. For those who may not be able to use and benefit from technology for a variety of reasons including cognitive, mental, and physical/functional health challenges, alternative methods (e.g., telephone-based services including dedicated hotlines, home visits, and caregiver training and support) of delivering equitable healthcare and social services and enhancing social integration are also needed.

## Conclusions

Email/texting has become an essential tool for healthcare-related communications, and telehealth remains an important post-pandemic healthcare delivery tool. Both email/texting and online tools can provide homebound older adults with a means to stay connected with family, friends, and other social networks. However, our findings show that even among those who own an ICT device, homebound older adults were less likely to use ICT. Concerted efforts are needed to remedy digital inequities among homebound older adults.
